# Four Subgroups of Blood Stasis Syndrome Are Identified by Manifestation Cluster Analysis in Males

**DOI:** 10.1155/2019/2647525

**Published:** 2019-07-08

**Authors:** Sooseong You, Byoung-Kab Kang, Jihyun Kim, Hoyoung Lee, Eun Hyoung Shim, Mi Mi Ko, Jiae Choi, Tae-Young Choi, Ji Hee Jun, Jeeyoun Jung, Minho Cha, Ju Ah Lee, Myeong Soo Lee

**Affiliations:** ^1^Korea Medicine Fundamental Research Division, Korea Institute of Oriental Medicine, Daejeon, Republic of Korea; ^2^Medical Research Division, Korea Institute of Oriental Medicine, Daejeon, Republic of Korea

## Abstract

Blood stasis syndrome (BSS) is an important pathological condition in traditional East Asian medicine and is associated with ischemic heart disease, cerebral vascular accident, diabetes mellitus, chronic renal failure, severe traumatic injury, and dysmenorrhea. However, previous studies have been unable to reveal the clinical and biological characteristics or biological markers of BSS. We hypothesized that the heterogeneity among the manifestations of BSS or non-BSS could interfere with an analysis to describe the characteristics of BSS. In this study, male participants based on the severity of BSS-associated symptoms and signs were clustered and classified into four subgroups: BSS subgroups (1), (2), (3), and (4). Non-BSS core subgroup was redefined using manifestation cluster analysis. Biological characteristics of subgroups BSS(1) and BSS(2) belong to the range of the non-BSS core subgroup (1), whereas that of subgroups BSS(3) and BSS(4) are characterized by different biological parameters such as systemic inflammatory conditions and elevated D-dimer level. Our results suggested that patients in subgroups of BSS(3) and BSS(4) are more likely to be exposed in an inflammatory state than other BSS subgroups. We found the heterogeneity among the manifestations which could mask the characteristics of BSS and identified the clinical and biological profiles of the four BSS subgroups through comparisons of the redefined non-BSS and BSS subgroups. This finding could provide accurate diagnostic criteria and new approaches for BSS treatments in different subgroups.

## 1. Introduction

Blood stasis is a pathological concept in traditional East Asian medicine that refers to stagnant blood that has lost its physiological function within the body [1–3] and leads to blood stasis syndrome (BSS), which is characterized by multiple signs and symptoms, such as sublingual varicosis, angiotelectasis, a slow and choppy pulse, local fixed pain, nyctalgia, menstrual cramps, a dark-purple tongue, or infraorbital darkness [2, 4]. Clinical studies have reported that these manifestations are observed in patients with ischemic heart disease, cerebral vascular accident, diabetes mellitus, chronic renal failure, severe traumatic injury, and dysmenorrhea [3, 5]. In addition, traditional herbal formulas for BSS were effective in relieving the severity of these diseases [6–10]. However, previous studies could not determine the clinical and biological characteristics or biological markers of BSS [5, 6, 10–12].

In psychoneurological filed, many studies have clustered pattern of symptoms with a psychoneurological symptom cluster intensity score because they have showed high heterogeneity which lead diagnosis and therapeutic failures [13, 14]. Clustering analysis is a method to define subgroups of individuals with high heterogeneity to explore clinical phenotypes in patients with various diseases [15]. Classification of disease into subtypes which have different clinical signs in terms of prognosis and individual differences might be needed to explain between clinical phenotype and biological mechanisms [16].

To overcome the limitations of previous studies, we diagnosed and classified into subgroups of non-BSS and BSS participants based on BSS-associated manifestation cluster analysis. We found that the heterogeneity among the manifestations of BSS within individuals could be considered to be an independent parameter for determining the characteristics of BSS. This approach identified subgroup-specific clinical characteristics and could lead to other studies on the biological markers of BSS.

## 2. Materials and Methods

### 2.1. Study Design

This was a community-based, multicenter trial that was designed as a cross-sectional observational study to identify the biological characteristics of BSS. The geographic data of the eligible participants were collected, and two Korean medicine doctors (KMD) independently estimated the severity of the clinical symptoms and signs of BSS as well as made a diagnosis of BSS or non-BSS for each participant. Finally, blood sampling was performed for the biological analysis. The detailed study protocol was presented to [17] and approved by the Institutional Review Board of the Korea Institute of Oriental Medicine (IRB no. I-1310/001-001-01) as well as the seven participating medical centers. The protocol used in this study was registered at Clinical Research Information Service (CRIS, register number KCT0000916) in Korea which is one of the primary registries of the WHO International Clinical Trials Registry Platform.

### 2.2. Participants

Six hundred seven inpatients and outpatients who met the eligibility criteria for this study were enrolled in the following seven traditional Korean medicine hospitals from July 2013 to December 2013: Kyung Hee Oriental Medicine Center, Kyung Hee University Oriental Hospital at Gangdong, Won Kwang Oriental Medical Hospital, Jaseng Hospital of Oriental Medicine, Cha Medical Center, and Pusan National University Korean Medicine Hospital. We utilized the data of 476 participants with the same diagnosis from two KMDs to guarantee the reliability of the diagnoses. However, we could not enroll enough female participants to perform manifestation cluster analysis with meaningful results. Female data could not be combined with male data because of the differences in clinically important symptoms, such as menstrual cramps and lumps in menstrual blood. Therefore, we excluded female participants from this analysis. The data from 219 of male participants were enrolled for a cluster analysis (supplementary Figure 1).

The eligibility criteria were as follows: males aged between 20 and 70 years who gave their written informed consent to participate and agreed to comply with the study regulations. The exclusion criteria were patients with any psychiatric condition that rendered them unable to communicate, critically ill patients, pregnant women, or patients with any conditions that could influence the study assessment.

### 2.3. Assessment of Clinical Symptoms and Signs

The KMD was trained twice with the standard operating procedures to estimate the severity of 31 BSS manifestations including discoloration within the body, local pain and tenderness, and disorder of blood circulation for accurately diagnosis. Thirty-one indicators for BSS were derived through 3 meetings by an expert committee in three countries: Korea, China, and Japan [3, 18, 19]. And two KMDs independently scored the participants using the “case report form for the diagnostic technology of blood stasis questionnaire-1”. Scores were given according to the following scale: 1 = none, 2 = slight, 3 = moderate, 4 = severe, and 5 = very severe. We used the average scores of individual variables for the analysis.

### 2.4. Analysis of the Biological Parameters

Blood was collected from each participant at the hospitals and transported to the Samkwang Medical Laboratory (Seoul, Korea) for analysis.

### 2.5. Statistical Analysis

All statistical analyses were performed using the Statistical Analysis System (SAS version 9.1.3, SAS Institute Inc., Cary, NC, USA). All P-values were two-sided, and P < 0.01 was considered statistically significant. Continuous variables were expressed using the mean ± standard deviation (SD), and categorical variables, such as smoking, drinking, and medication usage, were described by a number (percentage; Table 1). The significance of the differences in the general characteristics between groups was calculated using one-way analysis of variance (ANOVA) or the Chi-square test. To identify subgroups of participants based on their BSS manifestations, we carried out Ward's Minimum Variance Cluster analysis with Eigenvalue and pseudo T-squared statistics and determined the number of subgroups as well as used hierarchical clustering with squared Euclidean distances [20, 21]. Differences in the manifestations and 34 biological parameters between the redefined non-BSS and BSS subgroups were examined by the independent t-test or Wilcoxon Rank Sum test (Table 2).

## 3. Results

### 3.1. Manifestation Heterogeneity of the BSS and Non-BSS Participants

**Figure 1:**
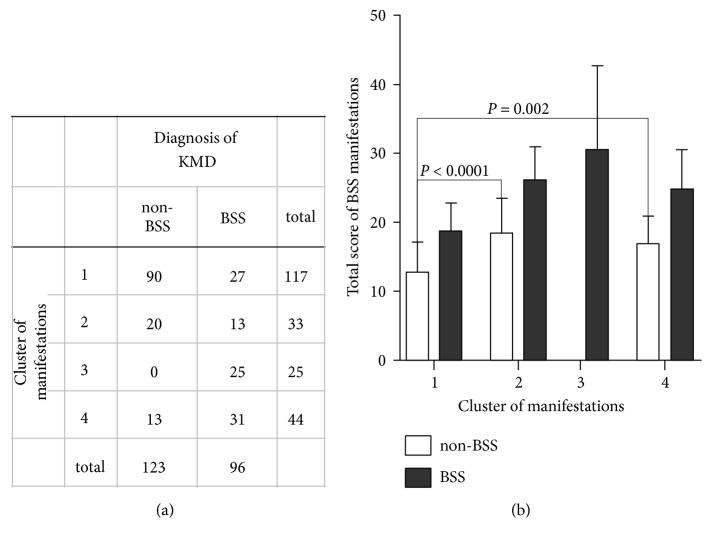
*Manifestation clustering and diagnoses of the enrolled participants*. (a) Participants with BSS or non-BSS were clustered based on BSS manifestations. The numbers of enrolled participants in the subgroups are shown. (b) Each bar represents the mean ± standard deviation of the average total score of each BSS manifestation. The total score is calculated by subtracting 31 from the sum of each original variable score. The* P*-value was calculated using an independent t-test.

We clustered male participants based on the severity of their BSS-associated symptoms and signs to reveal the manifestation heterogeneity among the BSS and non-BSS groups. A diagnosis with non-BSS included three manifestation clusters, and participants with BSS were divided into four subgroups (Figure 1(a)), showing that the manifestation heterogeneity of diagnosis could mask the clinical and biological profile of BSS. BSS and non-BSS participants classified in the same cluster could have substantial similarities in their biological characteristics.

### 3.2. The Redefined Non-BSS Core and Four Subgroups of BSS

To overcome the limitations of previous studies that did not consider the masking effect of manifestation heterogeneity, we redefined the cluster 1 group with non-BSS as a core of non-BSS, which had 73.1% (90/123) of participants with non-BSS (Figure 1(a)) and the lowest scores for BSS manifestations (Figure 1(b), p < 0.001). Participants with BSS were divided into four subgroups. The non-BSS core and subgroups of BSS are represented by diagnosis with clustering numbers as BSS (1) to BSS (4), and the general characteristics of the groups are presented in Table 1. The non-BSS(1) group had a lower proportion of participants who smoke, but age, BMI, SBP, DBP, drinking status, and medication were similar among the five groups (Table 1).

### 3.3. Differences in the Manifestations between the Redefined Non-BSS and BSS Subgroups

**Figure 2:**
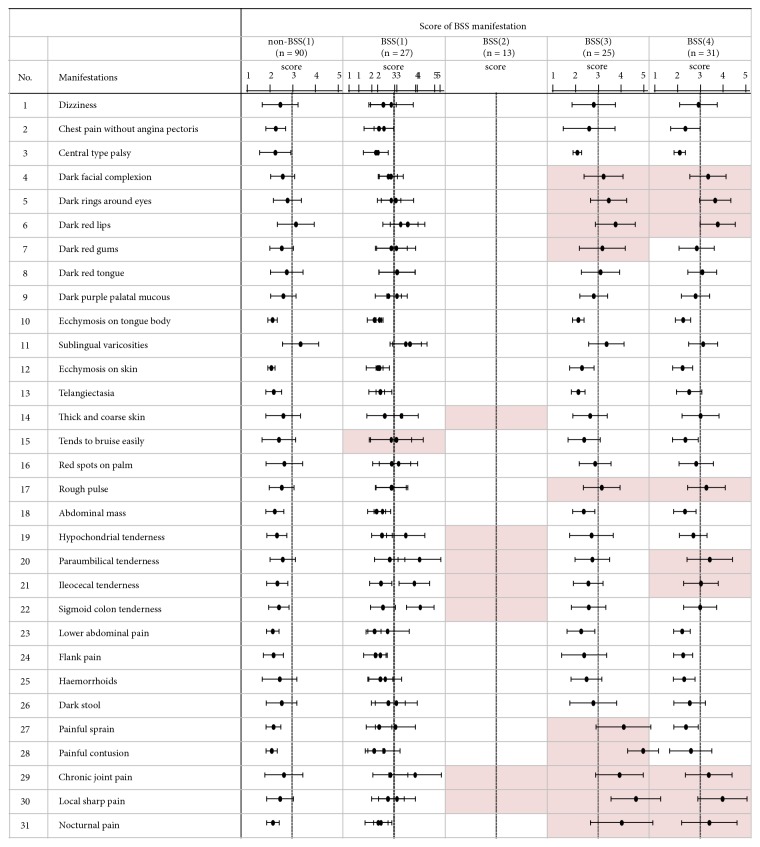
*Differences in manifestations between the redefined non-BSS and BSS subgroups*. Each bar represents the mean ± standard deviation of the average BSS manifestation score. A filled box represents a mean score ≥ 3 and a* P*-value < 0.01 compared to the non-BSS(1) group.

We identified BSS subgroup-specific clinical symptoms and signs through a comparison of BSS manifestation severities between non-BSS(1) and BSS subgroups. Subgroup BSS(1) had a tendency to bruise easily, and subgroup BSS(2) had abdominal tenderness, chronic joint pain, and local sharp pain (Figure 2, p < 0.01). Subgroups BSS(3) and BSS(4) commonly exhibited a rough pulse, chronic pain, local sharp pain, nocturnal pain, and discoloration of the face and under the eyes and lips (Figure 2, p < 0.01). In particular, participants of subgroup BSS(3) suffered from painful sprains and contusions (Figure 2, p < 0.01). These results suggest that each subgroup of BSS is characterized by different clinical symptoms and signs.

### 3.4. The Differences in the Biological Parameters between the Redefined Non-BSS and BSS Subgroups

Biological characterization of the BSS subgroups was performed through a comparison of the biological parameters between the non-BSS(1) and BSS subgroups. Subgroups BSS(1) and BSS(2) were not different from non-BSS(1) relative to our biological parameters, except for AST level (Table 2). Subgroups BSS(3) and BSS(4) were commonly characterized by an increased WBC count, percentage of neutrophils, and D-dimer level and decreased percentage of lymphocytes. Subgroup BSS(3) differentially expresses decreased RBC and albumin level and increased CRP level compared to those in subgroup BSS(4) (Table 2, p < 0.01). These results suggest that subgroups BSS(1) and BSS(2) belong to the range of the non-BSS(1) group and that subgroups BSS(3) and BSS(4) are characterized by different biological parameters.

## 4. Discussion

To identify the significant clinical and biological characteristics, data of enrolled participants were analyzed using Ward's Minimum Variance Cluster analysis with Eigenvalue and CCC. Firstly, male BSS participants were classified into 2, 4, or 6 clusters, and clinically relevant phenotypes have been identified in 4 clusters with Eigenvalue ≥ 1, which indicate positive definite to further analysis. As shown in Figure 1, we found four subgroups of BSS and non-BSS core group based on the severity of their BSS-associated manifestations using cluster analysis. The almost BSS manifestation severity of subgroup BSS(1) belongs to the range of the non-BSS(1) group, and subgroup BSS(2) exhibits abdominal tenderness and chronic pain (Figure 2). However, these subgroups are not biologically different from the non-BSS(1) group (Table 2). Therefore, subgroups BSS(1) and BSS(2) should be excluded from analysis to identify biological characteristics and markers of BSS. Subgroups BSS(3) and BSS(4) are commonly characterized by a rough pulse, discoloration of the skin, chronic pain, and increased D-dimer level. The BSS(3) subgroup is distinguished from the other subgroups by traumatic pain, decreased RBCs and albumin level, and increased CRP level. Further clinical studies on subgroups BSS(3) and BSS(4) are necessary to evaluate the effectiveness of BSS-specific treatments based on these biological parameters.

The symptoms and signs of BSS is correlated with pathologic properties of senescent RBCs [1]. Accelerated RBC senescence could induce low levels of hemoglobin and hematocrit in subgroups of BSS(3). Also, the accumulation of senescent RBCs causes thrombosis and blood clotting. The D-dimer level is elevated in patients with blood clotting disorders, and it increased the risk for thrombosis [22, 23]. Neutrophils and monocytes play a role in thrombus formation, and it contributes thrombo-inflammatory conditions [24]. Systematic inflammatory condition may elevate the level of CRP which is shown in subgroups of BSS(3) [25]. Therefore, according to our data, patients in subgroups of BSS(3) and BSS(4) might be more susceptible to develop severe diseases than those of BSS(1) and BSS(2). However, the prospective cohort study needs to prove prognosis of diseases depending on BSS subgroups.

When comparing two noncore subgroups of non-BSS(non-BSS(2), non-BSS(4)) with two subgroups of BSS(2) and BSS(4), biological characteristics were not different, except for WBC count in the comparison between non-BSS(4) and BSS(4) subgroups (Supplementary Table 1). It may mask the biological profiles of patients with BSS. Identification of clinical and biological profiles in a BSS needs to be considered the heterogeneity of manifestations in further studies.

Participants in four BSS subgroups smoked more cigarettes than non-BSS core group as shown in Table 1 (p < 0.05). People who smoke have showed increased erythrocyte sedimentation rate (ESR) which is a certain biomarker of inflammation [26]. Elevated ESR level has correlation with raised D-dimer level in patients with thrombosis and hemostasis [27]. Smoking habit may be a potential risk marker for blood stasis.

Although intense clinical studies are still necessary to identify the biological characteristics and markers of BSS, this new approach was able to verify the heterogeneity among the manifestations of a BSS diagnosis that could mask the clinical and biological profiles of BSS and identify four subgroups of BSS in males.

## 5. Conclusions

We verified the heterogeneity among the manifestations of a BSS diagnosis that could mask the clinical and biological profiles of BSS and identify four subgroups of BSS in males. This new approach could lead to other studies on the pathologic mechanism and biomarker of BSS.

## Figures and Tables

**Table 1:** General characteristics of the enrolled participants.

	Diagnosis (manifestation clustering number)					
Variables	non-BSS(1)	BSS(1)	BSS(2)	BSS(3)	BSS(4)	*P*-value
	(N=90)	(N=27)	(N=13)	(N=25)	(N=31)	
Age (year)	43.8 ± 11.8	48.5 ± 9.6	43.2 ± 12.2	47.2 ± 9.2	44.3 ± 11.6	0.271
BMI (kg/m^2^)	24.5 ± 2.9	24.4 ± 2.7	25.5 ± 2.6	24.5 ± 2.9	24.0 ± 2.8	0.567
SBP (mmHg)	121.5 ± 13.9	123.4 ± 16.1	128.3 ± 12.4	129.1 ± 12.0	123.0 ± 12.9	0.111
DBP (mmHg)	76.7 ± 10.5	77.7 ± 12.9	79.0 ± 8.3	83.2 ± 7.3	76.8 ± 8.3	0.072
Smoking (n, %)	25 (27.8)	11 (40.7)	7 (53.8)	15 (60.0)	14 (45.2)	0.025
Drinking (n, %)	56 (62.2)	16 (59.3)	9 (69.2)	11(44.0)	17 (54.8)	0.482
On medication (n, %)	48 (53.3)	15 (55.6)	6 (46.2)	19 (76.0)	22 (71.0)	0.147

The data are expressed as the mean ± standard deviation or a number with a frequency. BMI: body mass index; SBP: systolic blood pressure; DBP: diastolic blood pressure. The *P*-value was calculated using an ANOVA or the Chi-square test.

**Table 2:** Differences in the biological parameters between the redefined non-BSS and BSS subgroups.

		non-BSS	BSS			
No.	Biologic parameter, median (Q1-Q3)	non-BSS(1)	BSS(1)	BSS(2)	BSS(3)	BSS(4)
		(n = 90)	(n = 27)	(n = 13)	(n = 25)	(n = 31)
1	RBC (10^6^/*μ*L)	4.77 (4.52-5.04)	4.77 (4.48-5.02)	4.92 (4.65-5.08)	**4.5 (4.13-4.82)**	4.85 (4.51-5.26)
2	Hb (g/dL)	14.7 (14.2-15.5)	14.8 (14.1-15.6)	14.9 (14.7-15.5)	**14 (12.3-14.9)**	15.3 (14.3-16.3)
3	Hct (%)	44.15 (41.6-45.7)	44 (41.9-46.1)	43.7 (42.8-46.1)	**42.7 (39.4-44.8)**	45.7 (42-46.9)
4	MCV (fL)	92.35 (90-94.5)	91.7 (89.1-94.7)	91 (90-94.6)	92.9 (89.7-96.5)	92.1 (89.5-94)
5	MCH (pg)	31.1 (30.3-31.9)	31.4 (30.1-31.7)	31.2 (30.7-31.3)	31.1 (29.9-32.2)	31.3 (30.5-32)
6	MCHC (%)	33.8 (33-34.4)	33.9 (33.3-34.4)	33.8 (33.4-34.8)	33.3 (32.5-34)	34 (33.5-34.5)
7	RDW (%)	12.8 (12.5-13.3)	12.9 (12.5-13.3)	12.9 (12.7-13.3)	13.4 (12.6-14.2)	12.9 (12.4-13.3)
8	Platelet (10^6^/*μ*L)	223.5 (185-253)	234 (216-275)	227 (193-280)	22.8 (188-264)	23.8 (215-263)
9	MPV (fL)	10.7 (10.2-11.3)	10.3 (10-11)	10.7 (10.3-11.1)	10.6 (10.2-11)	10.9 (10.2-11.6)
10	PDW (%)	12.05 (11.1-13.1)	11.1 (10.8-12.4)	11.9 (11.1-12.8)	11.9 (10.7-12.7)	12.4 (11.1-14.1)
11	WBC (10^3^/*μ*L)	5.37 (4.68-6.62)	5.71 (4.66-7.27)	5.53 (4.36-7.21)	**6.62 (6.15-7.93)**	**6.56 (5.95-8.19)**
12	Neutrophil (%)	51.55 (48.6-58.5)	52.5 (47.2-57.4)	48.8 (44.8-55.1)	**59.4 (53.7-64.3)**	**57.5 (52.6-62.9)**
13	Lymphocyte (%)	35.8 (29.6-40.5)	36.3 (31.2-39.8)	41.9 (37.2-46.3)	**30 (24.1-37.1)**	**30.1 (25.3-34.2)**
14	Monocyte (%)	7.15 (6.3-8.8)	7.7 (6.6-8.7)	7.8 (5.7-8.7)	7.2 (6.2-10)	7.4 (6.2-9.2)
15	Eosinophil (%)	2.5 (1.9-4.6)	2.5 (1.5-5.1)	2.6 (0.9-4.3)	2.1 (1.2-3.4)	2.3 (1.6-4.5)
16	Creatinine (mg/dL)	0.93 (0.85-1.03)	0.9 (0.86-0.98)	0.91 (0.85-1)	0.89 (0.82-0.95)	0.9 (0.85-0.98)
17	BUN (mg/dL)	13 (11.4-15.3)	14.2 (13-16.8)	11.9 (10.4-15.4)	11.6 (9.5-15.3)	13.9 (11.5-16.7)
18	Total protein (g/dL)	7.02 (6.68-7.31)	7.02 (6.7-7.25)	7.15 (6.87-7.27)	6.84 (6.52-7.12)	7.04 (6.67-7.29)
19	Total cholesterol (mg/dL)	169.5 (149-206)	174 (151-210)	172 (150-187)	174 (155-186)	169 (161-197)
20	HDL(mg/dL)	49.7 (39.9-59.1)	46.3 (40.7-56.8)	46.3 (32.4-53.9)	43.2 (34-55.7)	45.5 (39-53.6)
21	Triglyceride (mg/dL)	136.5 (89-201)	133 (91-218)	165 (127-286)	150 (104-246)	153 (109-200)
22	Total lipid (mg/dL)	485.5 (422-581)	486 (454-614)	525 (443-619)	520 (437-567)	487 (422-598)
23	AST (IU/L)	19 (16-23)	19 (17-26)	**23 (21-25)**	21 (16-23)	21 (18-29)
24	ALT (IU/L)	18.5 (14-25)	21 (16-34)	27 (20-28)	20 (15-26)	25 (16-31)
25	ALP (IU/L)	63.5 (54-71)	63 (57-80)	61 (54-69)	73 (61-81)	60 (54-73)
26	Total bilirubin (mg/dL)	0.39 (0.3-0.56)	0.44 (0.26-0.6)	0.41 (0.36-0.54)	0.38 (0.23-0.57)	0.43 (0.3-0.59)
27	Direct bilirubin (mg/dL)	0.19 (0.13-0.25)	0.18 (0.12-0.23)	0.19 (0.14-0.24)	0.18 (0.11-0.22)	0.17 (0.13-0.24)
28	Indirect bilirubin (mg/dL)	0.2 (0.15-0.31)	0.2 (0.14-0.4)	0.2 (0.14-0.32)	0.18 (0.12-0.38)	0.25 (0.17-0.35)
29	Albumin (g/dL)	4.5 (4.33-4.7)	4.44 (4.32-4.7)	4.6 (4.44-4.74)	**4.3 (4.14-4.53)**	4.43 (4.29-4.6)
30	Globulin (g/dL)	2.51 (2.32-2.71)	2.47 (2.29-2.74)	2.45 (2.41-2.55)	2.52 (2.35-2.67)	2.52 (2.29-2.71)
31	A/G ratio	1.8 (1.66-1.99)	1.83 (1.64-.1.94)	1.86 (1.79-1.99)	1.75 (1.55-1.82)	1.81 (1.61-1.93)
32	Fibrinogen (mg/dL)	256 (218-307)	247 (216-289)	239 (203-287)	281 (222-339)	276 (246-348)
33	D-dimer (*μ*g/mL)	0.2 (0.2-0.2)	0.2 (0.2-0.3)	0.2 (0.2-0.2)	**0.3 (0.2-0.6)**	**0.2 (0.2-0.3)**
34	CRP (mg/L)	0.5 (0.3-1.1)	0.5 (0.3-1.5)	0.6 (0.4-1)	**1.7 (0.4-8.4)**	0.7 (0.4-1.7)

A bold font represents a *P* value < 0.01 compared to the non-BSS(1) group using Wilcoxon Rank Sum test. RBC: red blood cell; Hb: hemoglobin; Hct: hematocrit; WBC: white blood cell; CRP: C-reactive protein.

## Data Availability

The data used to support the findings of this study are available from the corresponding author upon request.

## Abbreviations

BMI: Body mass index
BSS: Blood stasis syndrome
CRP: C-reactive protein
DBP: Diastolic blood pressure
Hb: Hemoglobin
Hct: Hematocrit
OMD: Oriental medicine doctor
RBC: Red blood cell
SBP: Systolic blood pressure
WBC: White blood cell.

## Supplementary Materials

Supplementary Materials
* Supplementary Figure 1: flow diagram of patient enrollment*. The flow diagram in Supplementary Figure 1 describes the patient enrollment. The data from 219 male participants with same diagnosis by two physicians were analyzed to identify the biological characteristics of BSS. The female data were excluded in this study.* Supplementary Table 1: differences in the biological parameters between the non-BSS and BSS subgroups*. A filled box represents a* P*-value < 0.01 in two comparisons; non-BSS(2) vs. BSS(2), and non-BSS(4) vs. BSS(4). RBC: red blood cell; Hb: hemoglobin; Hct: hematocrit; WBC: white blood cell; CRP: C-reactive protein.
