# Supplementary material for: Four Subgroups of Blood Stasis Syndrome Are Identified by Manifestation Cluster Analysis in Males
Source: Evid Based Complement Alternat Med. 2019 Jul 8;2019:2647525. doi: 10.1155/2019/2647525 (PMC6644214; doi:10.1155/2019/2647525)
Supplement: Supplementary Materials — Supplementary Figure 1: flow diagram of patient enrollment. The flow diagram in Supplementary Figure 1 describes the patient enrollment. The data from 219 male participants with same diagnosis by two physicians were analyzed to identify the biological characteristics of BSS. The female data were excluded in this study. Supplementary Table 1: differences in the biological parameters between the non-BSS and BSS subgroups. A filled box represents a P-value < 0.01 in two comparisons; non-BSS(2) vs. BSS(2), and non-BSS(4) vs. BSS(4). RBC: red blood cell; Hb: hemoglobin; Hct: hematocrit; WBC: white blood cell; CRP: C-reactive protein. [file 2647525.f1.docx]

**Supplementary Table 1. Differences in the biological parameters between the non-BSS and BSS subgroups**

|  |  | **non-BSS** | | **BSS** | |
| --- | --- | --- | --- | --- | --- |
| **No.** | **Biologic parameter,**  **median (Q1-Q3)** | **non-BSS(2)**  **(n = 20)** | **non-BSS(4)**  **(n = 13)** | **BSS(2)**  **(n = 13)** | **BSS(4)**  **(n = 31)** |
| **1** | **RBC (10^6^/μL)** | 4.84 (4.55-5.03) | 5.09 (4.66-5.25) | 4.92 (4.65-5.08) | 4.85 (4.51-5.26) |
| **2** | **Hb (g/dL)** | 14.75 (14.1-15.2) | 15.5 (14.5-15.8) | 14.9 (14.7-15.5) | 15.3 (14.3-16.3) |
| **3** | **Hct (%)** | 43.35 (42.5-45.5) | 45.3 (43.2-46.9) | 43.7 (42.8-46.1) | 45.7 (42-46.9) |
| **4** | **MCV (fL)** | 91.15 (88.65-94.55) | 90.1 (87.5-92.7) | 91 (90-94.6) | 92.1 (89.5-94) |
| **5** | **MCH (pg)** | 30.95 (30.05-31.7) | 30.3 (29.9-31) | 31.2 (30.7-31.3) | 31.3 (30.5-32) |
| **6** | **MCHC (%)** | 33.75 (32.5-34.65) | 33.8 (33.4-34.6) | 33.8 (33.4-34.8) | 34 (33.5-34.5) |
| **7** | **RDW (%)** | 12.9 (12.45-13.45) | 12.7 (12.6-13.1) | 12.9 (12.7-13.3) | 12.9 (12.4-13.3) |
| **8** | **Platelet (10^6^/μL)** | 200 (177-245) | 228 (200-249) | 227 (193-280) | 23.8 (215-263) |
| **9** | **MPV (fL)** | 11.2 (10.55-11.9) | 10.9 (10.5-11.3) | 10.7 (10.3-11.1) | 10.9 (10.2-11.6) |
| **10** | **PDW (%)** | 13.45 (11.75-14.45) | 12.6 (12.3-13.3) | 11.9 (11.1-12.8) | 12.4 (11.1-14.1) |
| **11** | **WBC (10^3^/μL)** | 6.56 (6.09-7.23) | 5.86 (5.01-6.15) | 5.53 (4.36-7.21) | 6.56 (5.95-8.19) |
| **12** | **Neutrophil (%)** | 56.4 (51.75-63.25) | 56.3 (48.1-58.8) | 48.8 (44.8-55.1) | 57.5 (52.6-62.9) |
| **13** | **Lymphocyte (%)** | 32.15 (27.55-38.3) | 34.4 (30-39.4) | 41.9 (37.2-46.3) | 30.1 (25.3-34.2) |
| **14** | **Monocyte (%)** | 6.4 (5.5-8) | 7.6 (6.2-8.7) | 7.8 (5.7-8.7) | 7.4 (6.2-9.2) |
| **15** | **Eosinophil (%)** | 2 (1.3-3.95) | 3 (1.5-3.4) | 2.6 (0.9-4.3) | 2.3 (1.6-4.5) |
| **16** | **Creatinine (mg/dL)** | 0.9 (0.84-1.06) | 0.91 (0.84-1) | 0.91 (0.85-1) | 0.9 (0.85-0.98) |
| **17** | **BUN (mg/dL)** | 13.4 (11.6-16.25) | 12.5 (9.5-14.4) | 11.9 (10.4-15.4) | 13.9 (11.5-16.7) |
| **18** | **Total protein (g/dL)** | 7.06 (6.79-7.31) | 7.18 (6.9-7.32) | 7.15 (6.87-7.27) | 7.04 (6.67-7.29) |
| **19** | **Total cholesterol (mg/dL)** | 177.5 (141.5-189.5) | 177 (170-190) | 172 (150-187) | 169 (161-197) |
| **20** | **HDL(mg/dL)** | 45.7 (38.7-54.4) | 46.3 (40.4-51.8) | 46.3 (32.4-53.9) | 45.5 (39-53.6) |
| **21** | **Triglyceride (mg/dL)** | 152.5 (105-285) | 170 (137-236) | 165 (127-286) | 153 (109-200) |
| **22** | **Total lipid (mg/dL)** | 497.5 (387.5-571) | 524 (477-627) | 525 (443-619) | 487 (422-598) |
| **23** | **AST (IU/L)** | 23.5 (16-27.5) | 18 (15-24) | 23 (21-25) | 21 (18-29) |
| **24** | **ALT (IU/L)** | 23.5 (15.5-29.5) | 18 (16-26) | 27 (20-28) | 25 (16-31) |
| **25** | **ALP (IU/L)** | 60 (51.5-67.5) | 67 (52-70) | 61 (54-69) | 60 (54-73) |
| **26** | **Total bilirubin (mg/dL)** | 0.43 (0.29-0.57) | 0.35 (0.3-0.56) | 0.41 (0.36-0.54) | 0.43 (0.3-0.59) |
| **27** | **Direct bilirubin (mg/dL)** | 0.18 (0.13-0.23) | 0.17 (0.13-0.21) | 0.19 (0.14-0.24) | 0.17 (0.13-0.24) |
| **28** | **Indirect bilirubin (mg/dL)** | 0.24 (0.13-0.37) | 0.2 (0.15-0.33) | 0.2 (0.14-0.32) | 0.25 (0.17-0.35) |
| **29** | **Albumin (g/dL)** | 4.54 (4.33-4.77) | 4.61 (4.3-4.68) | 4.6 (4.44-4.74) | 4.43 (4.29-4.6) |
| **30** | **Globulin (g/dL)** | 2.54 (2.24-2.68) | 2.63 (2.5-2.68) | 2.45 (2.41-2.55) | 2.52 (2.29-2.71) |
| **31** | **A/G ratio** | 1.81 (1.69-1.98) | 1.74 (1.62-1.81) | 1.86 (1.79-1.99) | 1.81 (1.61-1.93) |
| **32** | **Fibrinogen (mg/dL)** | 247.5 (221.5-273.5) | 250 (231-287) | 239 (203-287) | 276 (246-348) |
| **33** | **D-dimer (μg/mL)** | 0.2 (0.2-0.2) | 0.2 (0.2-0.2) | 0.2 (0.2-0.2) | 0.2 (0.2-0.3) |
| **34** | **CRP (mg/L)** | 0.5 (0.3-1.25) | 0.5 (0.3-0.7) | 0.6 (0.4-1) | 0.7 (0.4-1.7) |

A filled box represents a *P* value < 0.01 in two comparisons; non-BSS(2) vs. BSS(2) and non-BSS(4) vs. BSS(4). RBC: red blood cell; Hb: hemoglobin; Hct: hematocrit; WBC: white blood cell; CRP: C-reactive protein.

Supplementary Figure 1. Flow diagram of patient enrollment


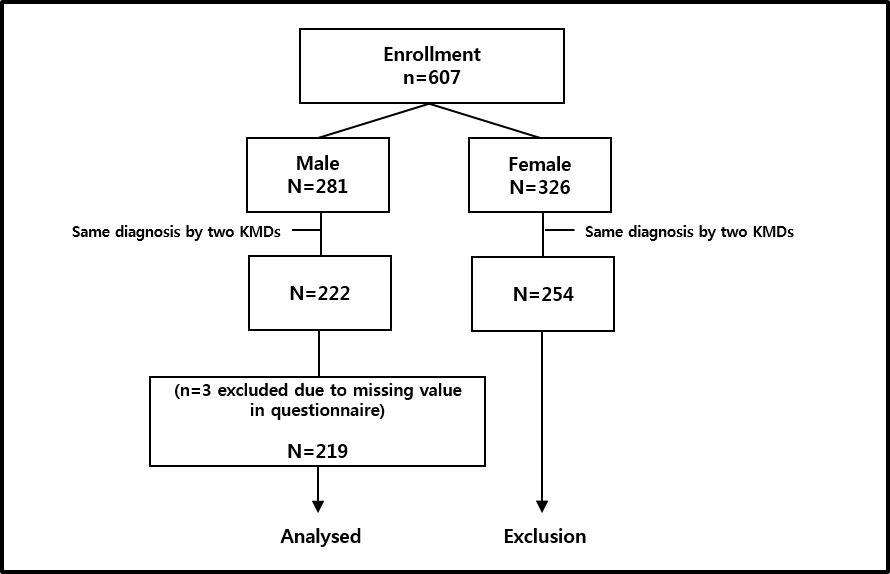


The flow diagram in supplementary figure 1 describes the patient enrollment. The data from 219 male participants with same diagnosis by two physicians were analyzed to identify the biological characteristics of BSS. The female data were excluded in this study.
